# Extraction and Surface Functionalization of Cellulose Nanocrystals from Sugarcane Bagasse

**DOI:** 10.3390/molecules28145444

**Published:** 2023-07-16

**Authors:** Sen Tang, Zhipeng Chen, Feifan Chen, Xuanren Lai, Qiaoyan Wei, Xianling Chen, Caiyun Jiang

**Affiliations:** 1 School of Food and Biochemical Engineering, Guangxi Science & Technology Normal University, Laibin 546199, China; ts540998577@163.com (S.T.);; 2 Guangxi Sugar Resources Engineering Technology Research Center, Guangxi Science & Technology Normal University, Laibin 546199, China; 3 Institute of Modern Cane Sugar Industry Development, Guangxi Science &Technology Normal University, Laibin 546199, China

**Keywords:** sugarcane bagasse, cellulose nanocrystals, preparation process, surface modification

## Abstract

The present study aimed to optimize the process for extracting cellulose nanocrystals (CNCs) from sugarcane bagasse through ultrasonic-assisted sulfuric acid hydrolysis and its subsequent modification with L-malic acid and silane coupling agent KH-550. The effects of the different modification methods and the order of modification on the structures and properties of bagasse CNCs were explored. The results indicated that the optimal process conditions were achieved at an acid-digestion temperature of 50 °C, a reaction time of 70 min, an ultrasonic power of 250 W, and a volume fraction of 55%. The modified CNCs were analyzed using infrared spectral, X-ray diffraction, and thermogravimetric techniques, which revealed that L-malic acid was attached to the hydroxyl group on the CNCs via ester bond formations, and the silane coupling agent KH-550 was adsorbed effectively on the CNCs’ surfaces. Moreover, it was observed that the modification of the CNCs by L-malic acid and the KH-550 silane coupling agent occurred only on the surface, and the esterification–crosslinking modification method provided the best thermal stability. The performance of self-made CNC was found to be superior to that of purchased CNC based on the transmission electron microscopy analysis. Furthermore, the modified esterified-crosslinked CNCs exhibited the best structure and performance, thereby offering a potential avenue for the high-value utilization of sugarcane bagasse, a byproduct of sugarcane sugar production, and the expansion of the comprehensive utilization of sugarcane bagasse.

## 1. Introduction

In the sugar industry, for every 1 t of cane sugar produced, 1 to 2 t of cane leaves, 2 to 3 t of bagasse, 800 kg of molasses, and 250 kg of sugarcane filter mud are generated [1]. Bagasse, as a byproduct produced during sugarcane processing, accounts for approximately 24% to 27% of the weight of sugarcane. Sugarcane bagasse has the characteristics of concentrated sources, high yield, and renewability, and it has great potential for utilization. However, currently, sugarcane bagasse is mainly burned or discarded by sugar mills as boiler fuel, with very low utilization efficiency. This has also led to the insufficient exploration of the comprehensive utilization value of sugarcane bagasse, resulting in resource waste and environmental pollution. The development and utilization of bagasse as a resource can promote the development of the sugar industry chain, and the high-value utilization of bagasse combined with the development of a green circular economy is a pressing issue that needs to be solved [2].

Cellulose nanocrystals (CNCs) are made from cellulose by hydrolysis and are characterized by their high crystallinity, low density, high specific surface area, and excellent optical properties. The variations in the size, morphology, and crystallinity of CNCs depend on the source of cellulose material and its preparation conditions [3,4]. In the past decade, cellulose nanocrystals have been further explored both domestically and internationally for a wide range of applications in biomedicine, wastewater treatment, energy, and electronics. CNCs are prepared using the following methods: strong acid hydrolysis [5], mechanical methods [6], biological enzyme hydrolysis [7], and TEMPO chemical oxidation [8]. Among these, the chemical method is the most conventional and simple preparation method, and it obtains cellulose largely through acid hydrolysis, but this method has certain limitations, with low efficiency being one of them [9]. In practice, the conventional acid hydrolysis method suffers from the incomplete hydrolysis of reactants, poorly carried-out hydrolysis, and difficult post-treatment, which greatly limits the large-scale production and application of cellulose nanocrystals. Therefore, the development of simple and efficient methods for the preparation of cellulose nanocrystals is necessary for the industrial production and application of CNCs.

Although the macromolecular chains of CNCs contain a large number of hydroxyl functional groups, the hydroxyl groups between the bagasse fibers are prone to forming hydrogen bonds. At the same time, bagasse fibers are prone to detachment during use, aggregation during processing, and poor dispersion. These characteristics limit the functionality and application range of CNCs, and so the chemical modification of CNCs is necessary. CNCs have commonly required chemical modification methods such as non-covalent bonding surface modification, esterification modification, silylation modification, cationic modification, and graft copolymerization modification [10,11,12,13,14]. Silylation methods have been widely used for the hydrophobic modification of CNCs, and silylation is a form of crosslinking modification. A coupling agent is able to combine nanoparticles and polymers using chemical reactions such as chemical bonding, and it has an amphoteric structure that allows it to also react with the hydroxyl groups on the surface of CNCs, thus not only improving the hydrophobicity of CNCs but also inhibiting the agglomeration of CNCs with surrounding CNCs while also improving their uniform dispersion in water [15]. Esterification modification is a common method used to improve the hydrophobicity of CNCs, which contain a large number of polar hydroxyl groups on their surfaces, and a variety of CNC esters can be generated by nucleophilic substitution reactions between the hydroxyl groups in the molecular chain and the acids, acyl halides, and anhydrides under the effect of a catalyst [16].

In this study, bagasse CNCs were prepared using an ultrasonic-assisted sulfuric acid hydrolysis method with bagasse as raw material, and the extraction process was optimized. Then, the CNCs were modified with L-neneneba malic acid and the silane coupling agent KH-550 using four methods: esterification, crosslinking, crosslinking esterification, and esterification crosslinking. The effects of the different modification methods and the order of modification on the structures and properties of bagasse CNCs were explored. This study provides new ideas for the high-value utilization of sugarcane bagasse.

## 2. Results and Discussion

### 2.1. Single-Factor Test Results

#### 2.1.1. Effect of Acid-Digestion Temperature on the Sugarcane Bagasse CNC Yield

**Figure 1:**
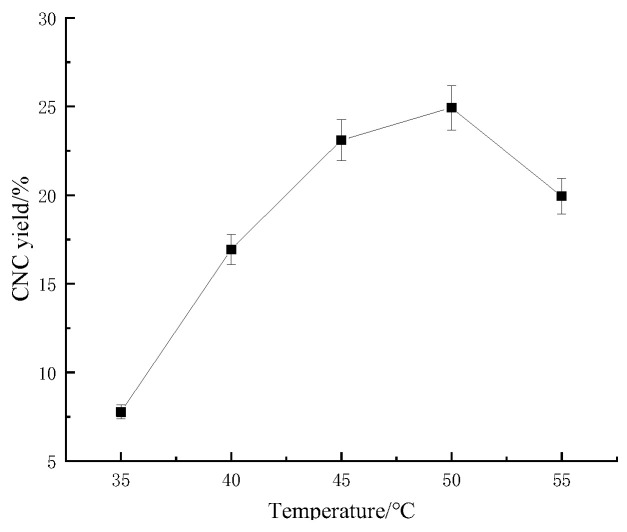
Effect of acid-digestion temperature on the sugarcane bagasse CNC yield.

As shown in Figure 1, with the increase in acid-digestion temperature, the CNC yield showed a trend of increasing and then decreasing, and the increase in temperature had a promoting effect on the CNC yield in a certain range, but a temperature beyond the given range destroyed the CNC stability and caused the CNC yield to decrease. The highest bagasse CNC yield (24.930%) was obtained when the reaction temperature was at 50 °C. This was because the acid hydrolysis reaction was insufficient at lower temperatures, and as the temperature increased, it promoted the breakage of the glycosidic bonds between the cellulose molecules, which increased nanoscale cellulose production and improved the CNC yield of the sugarcane bagasse. However, excessive temperatures again charred the cellulose and reduced the bagasse CNC yield [17].

#### 2.1.2. Effect of Acid-Digestion Time on the Sugarcane Bagasse CNC Yield

**Figure 2:**
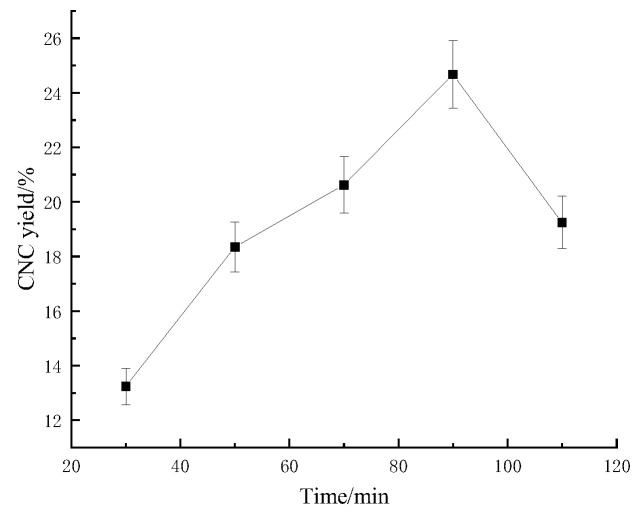
Effect of acid-digestion time on the sugarcane bagasse CNC yield.

As shown in Figure 2, with the increase in acid-digestion time, the CNC yield showed a trend of increasing and then decreasing, and the increase in acid-digestion time had a promoting effect on the CNC yield for a certain range. However, the CNC yield decreased after the acid-digestion time exceeded a certain range because the CNC structure was destroyed by the sulfuric acid. When the acid-digestion time was 90 min, the CNC yield was the highest (24.675%). This was because when the acid-digestion time was insufficient, the contact between the cellulose and the sulfuric acid was not sufficient, which led to insufficient breakage of the glycosidic bonds between the cellulose. With the increase in reaction time, the breakage of the glycosidic bonds between the cellulose was completed gradually. However, excessive reaction time led to the continued hydrolysis of the CNCs and degradation of the glucose, which decreased the bagasse CNC yield [18].

#### 2.1.3. Effect of the Volume Fraction of Sulfuric Acid on the CNC Yield of Sugarcane Bagasse

**Figure 3:**
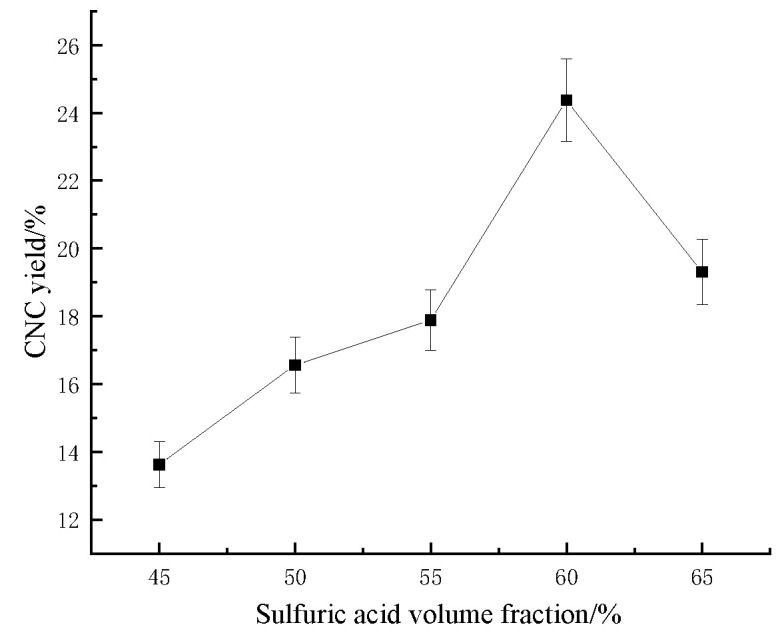
Effect of the sulfuric acid volume fraction on the sugarcane bagasse CNC yield.

As shown in Figure 3, with the increase in the sulfuric acid volume fraction, the CNC yield showed a trend of increasing and then decreasing, and the increase in the sulfuric acid volume fraction had a promoting effect on the CNC yield for a certain range. However, the sulfuric acid volume fraction over this range destabilized the CNC and caused the CNC yield to decrease. When the volume fraction of sulfuric acid was 60%, the highest bagasse CNC yield was achieved (24.370%). When the sulfuric acid concentration was low, the hydrogen ion concentration in the system was small, the degree of cellulose hydrolysis was low, and the amount of cellulose retained was high. As the sulfuric acid concentration was increased, the hydrogen ion concentration increased, and more cellulose intermolecular glycosidic bonds were broken, producing more nanoscale cellulose and increasing the bagasse CNC yield. However, excessive sulfuric acid concentration led to further hydrolysis of bagasse CNC, degradation to glucose, and even charring of the cellulose, further lowering the bagasse CNC yield.

#### 2.1.4. Effect of Acidolysis Ultrasonic Power on the Bagasse CNC Yield

**Figure 4:**
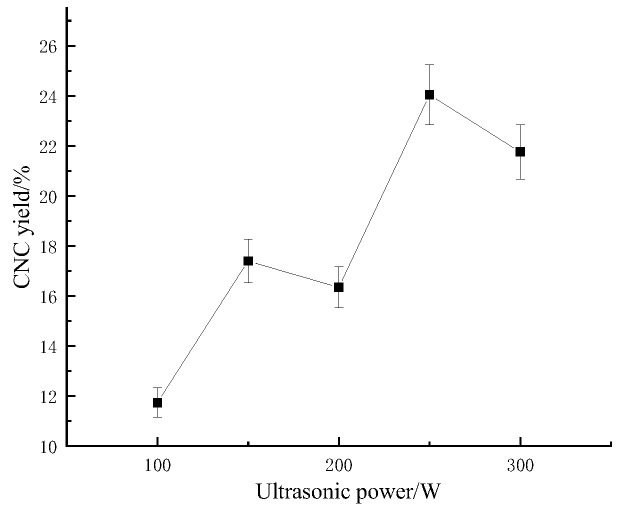
Effect of acidolysis ultrasonic power on the sugarcane bagasse CNC yield.

As shown in Figure 4, with the increase in the ultrasonic power of the acid digestion, the CNC yield of sugarcane bagasse showed an overall trend of first increasing, then decreasing, then increasing, and then decreasing. During the acid digestion, the ultrasonic waves mainly played the role of auxiliary sulfuric acid hydrolysis, which had the effect of promoting cellulose hydrolysis within a certain range. When the ultrasonic power reached 250 W, the CNC yield of sugarcane bagasse was the largest (24.040%). This was because, with the increase in ultrasonic power, the reaction process of the sulfuric acid hydrolysis of the cellulose was accelerated, which broke more of the glycosidic bonds between the cellulose molecular chains, but with the increase in power, excessive ultrasonic power further promoted the hydrolysis of the cellulose to glucose molecules, which decreased the sugarcane bagasse CNC yield.

### 2.2. Single-Factor Orthogonal Test Analysis

The single-factor orthogonal test results and analysis of the extreme differences is shown in Table 1.

The analysis of variance for the test results is shown in Table 2.

Through analysis of variance, it can be seen that factor D has no significant impact on the indicator, and the significance of the factor is also not high. Factor D can be incorporated into the error, that is, the fourth column of the orthogonal table can be used as the error column, that is, the sum of squared errors SSe = 0.413 + 7.140 = 7.553, and the degree of freedom of errors fe = 9 + 2 = 11. Then, significance tests can be performed on factors A, B, and C, as shown in Table 3.

The ANOVA after combining the errors showed the significance level of factor A, and because F_A_ = 49.392 > F_0.01_ (2, 11) = 7.20, factor A was significant at α = 0.01. For the significance level of factor B, because F_B_ = 2.933 > F_0.1_ (2, 11) = 2.86, factor B was significant at α = 0.1. For factor C’s significance level, because F_C_ = 47.990 > F_0.01_ (2,11) = 7.20, factor C was significant at α = 0.01. The effects of factors A, B, and C, i.e., acid-digestion temperature, acid-digestion time, and sulfuric acid volume fraction, on the extraction rates of sugarcane bagasse CNC were significant, and the significance levels of factors A, B, and C were increased. The effect of ultrasonic power on the index was not significant in the studied range, and the levels of the factors for the better combinations of parameters could be chosen arbitrarily.

From the extreme difference R, it could be seen that the order of each factor in the selected factor level was A > C > B > D. Then, through the combined error ANOVA with the better parameters in the single-factor test on the effect of acid-digestion ultrasonic power on the extraction rate of the sugarcane bagasse CNC, the best preparation process condition for sugarcane bagasse CNC was determined to be A_2_B_1_C_1_D_2_, i.e., the acid digestion was conducted at a temperature of 50 °C with a 55% volume fraction of sulfuric acid, and the acid digestion was carried out at a power of 250 W for 70 min. In the validation experiment, using the optimal conditions, the sugarcane bagasse CNC yield was 27.728 ± 0.4458, with RSD = 1.6078%, which indicated that the process conditions had good reproducibility.

### 2.3. CNC Modification and the Properties of the Modified CNCs

#### 2.3.1. TEM Analysis

**Figure 5:**
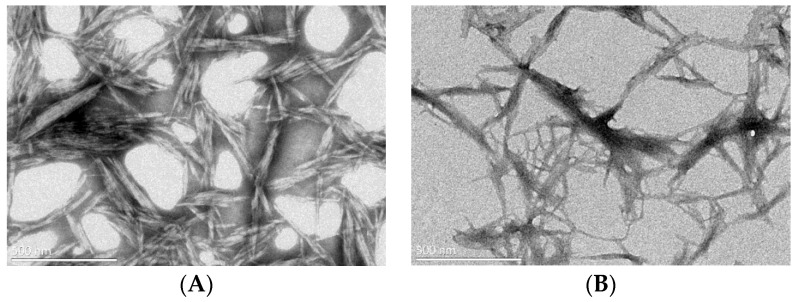
TEM diagrams of the self-made CNCs (**A**) and the purchased CNCs (**B**).

As shown in Figure 5, under transmission electron microscopy observation at 500 nm, the self-made CNCs had rod-like structures with lengths of 180–277 nm, diameters of 16–20 nm, and length-to-diameter ratios of 13–18. Although the purchased CNCs also had rod-shaped structures, their surfaces were rough, with lengths of 237–492 nm, diameters of 19–28 nm, and length-to-diameter ratios of 17–25. Compared to the purchased CNCs, the self-made CNCs had smaller sizes, which also indicated that self-made CNCs were more suitable for the experimental requirements.

#### 2.3.2. FTIR Analysis

**Figure 6:**
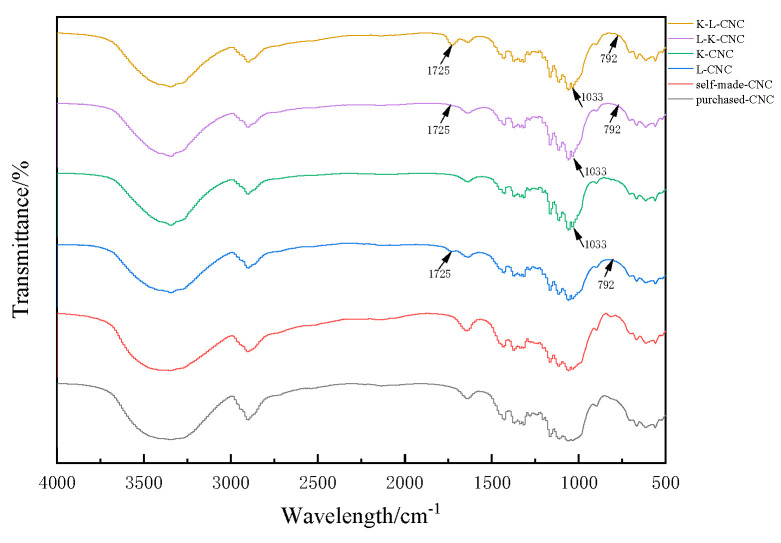
Infrared spectra of the K-L-CNC, L-K-CNC, K-CNC, L-CNC, purchased CNCs, and self-made CNCs.

As shown in Figure 6, the FTIR spectra of the K-L-CNC, L-K-CNC, K-CNC, L-CNC, self-made CNCs, and purchased CNCs were analyzed, and at a wavelength of 1725 cm^−1^, the K-L-CNC, L-K-CNC, and L-CNC showed new absorption peaks compared to the remaining three, which were was caused by the C=O stretching vibration in the L-malic acid. At a wavelength of 1033 cm^−1^, the K-L-CNC, L-K-CNC, and K-CNC showed new absorption peaks compared to the remaining three, which was due to the characteristic group of the silane coupling agent KH-550, indicating that the CNCs interacted with the silane coupling agent. At a wavelength of 792 cm^−1^, the absorption peaks of the K-L-CNC, L-K-CNC, and L-CNC compared with those of the K-CNC, self-made CNCs, and purchased CNCs disappeared, which was due to the esterification modification between the cellulose molecules and the L-malic acid, which caused the absorption peak to move to a wavelength of 1725 cm^−1^. Therefore, the esterification reaction occurred between the L-malic acid and the CNCs, and it was attached to the hydroxyl groups on the CNCs in the form of ester bonds. The silane coupling agent KH-550 interacted with the CNCs and caused the KH-550 to be well-adsorbed on the CNCs.

#### 2.3.3. XRD Analysis

**Figure 7:**
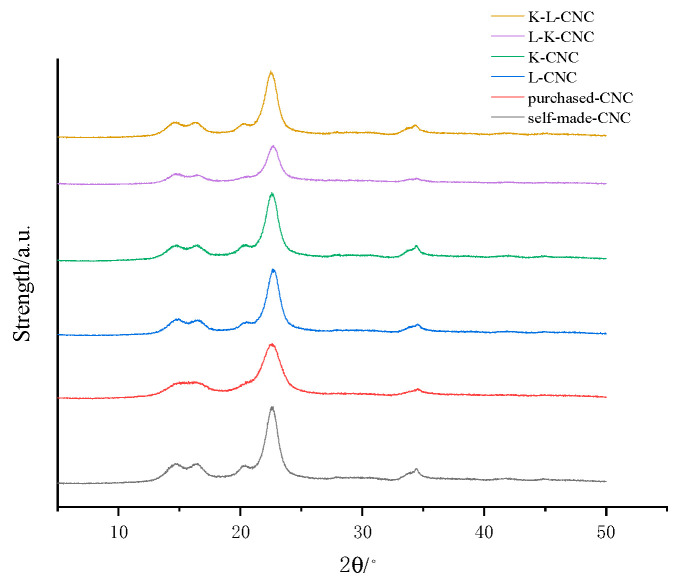
XRD analysis of the K-L-CNC, L-K-CNC, K-CNC, L-CNC, purchased CNCs, and self-made CNCs.

The X-ray diffraction analysis of the K-L-CNC, L-K-CNC, K-CNC, L-CNC, purchased CNCs, and self-made CNCs is shown in Figure 7. From the figure, it can be seen that the XRD diffraction peaks of the CNCs before and after modification were nearly the same, with the major diffraction peaks located at 14.7°, 16.6°, 22.5°, and 34.2°. The crystallinity indices of the K-L-CNC, L-K-CNC, K-CNC, L-CNC, purchased CNCs, and self-made CNCs were 88.778%, 82.884%, 87.904%, 87.575%, 88.321%, and 81.436%, respectively, based on the crystallinity index calculations. The crystallinity changed slightly after the CNC modification and appeared to increase, indicating that the modification of the CNCs by the L-malic acid and KH-550 silane coupling agent occurred only on the surfaces, and due to the action of the L-malic acid and KH-550 silane coupling agent on the CNCs, more crystalline regions were produced, which led to the increased crystallinity of the CNCs.

#### 2.3.4. TGA

**Figure 8:**
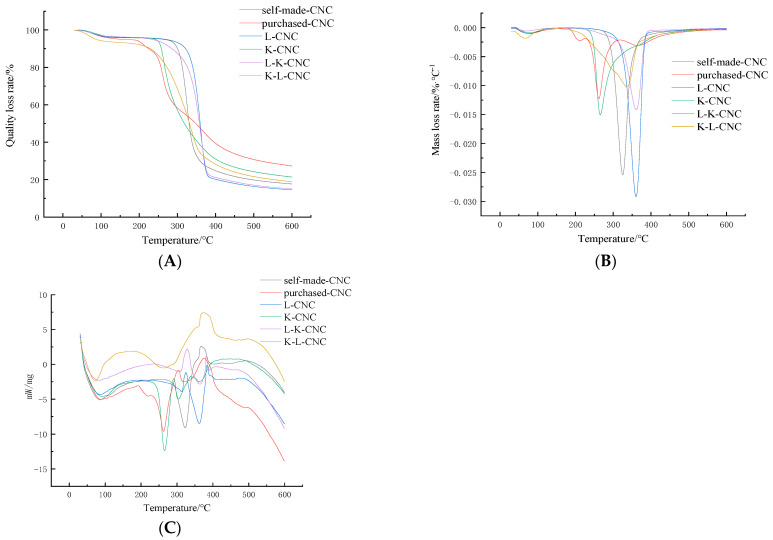
TGA: (**A**) DTG, (**B**) DSC, and (**C**) diagrams of the K-L-CNC, L-K-CNC, K-CNC, L-CNC, self-made CNCs, and purchased CNCs.

As shown in Figure 8, the initial decomposition temperatures and maximum weight-loss rate temperatures of the K-L-CNC, L-K-CNC, K-CNC, L-CNC, self-made CNCs, and purchased CNCs were 228 °C and 335 °C, 262 °C and 360 °C, 226 °C and 265 °C, 272 °C and 360 °C, 256 °C and 325 °C, and 214 °C and 262 °C, respectively. The maximum weight-loss rate temperatures increased for the self-made CNCs, the initial temperature and maximum weight-loss rate temperature of the K-CNC were lower than those of the self-made CNCs, and the initial decomposition temperature of the K-L-CNC was lower than that of the self-made CNCs, and so the thermal stability of the CNCs was improved after L-malic acid modification, while the thermal stability of the CNCs was reduced after the KH-550 silane coupling agent modification. When comparing the self-made CNCs with the purchased CNCs, we found that the thermal stability of the self-made CNCs was better than that of the purchased CNCs. The K-L-CNC, L-K-CNC, K-CNC, L-CNC, self-made CNCs, and purchased CNCs melted at temperatures of 371 °C, 328 °C, 290 °C, 325 °C, 366 °C, and 304 °C, respectively. This may have been due to the esterification reaction that reduced the hydroxyl groups on the surfaces of the CNCs, resulting in a decreased number of hydrogen bonds between the CNC molecules, which further reduced the thermal stability of the modified CNCs. Esterification led to decreases in the crystallinity of the surface structures of the modified CNCs. Therefore, the K-L-CNC had the best thermal stability.

## 3. Materials and Methods 

### 3.1. List of Materials 

The sugarcane bagasse was acquired from Guangxi Laibin Xianggui Sugar Co., Ltd. (Laibin, China). The sodium chlorite (AR), 95% ethanol, sodium hydroxide (AR), and sulfuric acid (AR) were bought from Chengdu West Asia Chemical Co., Ltd. (Chengdu, China), and the L-malic acid (RG) and silane coupling agent KH-550 were obtained from Shanghai Macklin Biochemical Co., Ltd. (Shanghai, China). CNCs were obtained from Kunshan Houpu Biofiber Technology Co., Ltd. (Suzhou, China).

### 3.2. Preparation of Bagasse Crude Cellulose

The preparation of the crude cellulose from sugarcane bagasse was based on the method of Luo Suqin, with slight modifications [19]. We added 50 g of dried sugarcane bagasse powder to distilled water and heated the mixture in a 75 ℃ water bath for 2 h. Then, the bagasse was washed with distilled water until the water was clear, and the water was drained with gauze, which was followed by a bleaching treatment for lignin removal. Then, a 7.5% sodium chlorite solution of pH 3.8–4.4 was added to the washed bagasse, and the reaction system was also bleached at 75 ℃ for 5 h. After the reaction, the bagasse was washed with water until the bagasse was pure white, and then the bagasse was washed and filtered with 95% ethanol to avoid cellulose agglomeration. It was then dried and set aside. Finally, the treated bagasse was mixed with 250 mL of 10% NaOH solution for an alkalization treatment to remove the hemicellulose and other impurities, and then it was washed with distilled water for the heavy suspension sedimentation to return to neutral. It was then dispersed with 95% ethanol for drying and then crushed and sieved to obtain 100-mesh-sieved bagasse coarse fiber with a white color.

### 3.3. Preparation of the Sugarcane Bagasse CNCs

The preparation of the bagasse CNCs was based on the method of Huang et al., with slight modifications [20]. The crude bagasse cellulose prepared as described above was dispersed in a specific volume fraction of sulfuric acid solution at a material-to-liquid ratio of 1:12 and fixed with different ultrasonic powers and temperatures, and then it was acid-digested for a certain time. The reaction was terminated by adding 10 times the volume of deionized water quickly after treatment and left to stand at room temperature for 12 h. We centrifuged the stationary sample at a speed of 10,000 r/min in a centrifuge, removed the supernatant, collected the bottom sediment, and repeated this process to return the sample’s pH to neutral. The light-yellow bagasse cellulose suspension was collected and transferred to a dialysis bag, where it was stored for 5 to 7 d. The dialysis was stopped when the pH of the suspension reached 6–7. The treated samples were dried in a vacuum freeze dryer to obtain the powdered bagasse CNCs.

### 3.4. Single-Factor Test for the Preparation of the Bagasse CNCs

The sugarcane bagasse CNCs were prepared according to the above method, and the effects of the acid-digestion temperature (35 ℃, 40 ℃, 45 ℃, 50 ℃, and 55 ℃), acid-digestion time (30 min, 50 min, 70 min, 90 min, and 110 min), sulfuric acid volume fraction (45%, 50%, 55%, 60%, and 65%), and ultrasonic power (100 W, 150 W, 200 W, 250 W, and 300 W) on the CNC yields were investigated. The fixed levels were as follows: acid-digestion temperature of 45 ℃, acid-digestion time of 70 min, sulfuric acid volume fraction of 55 %, and ultrasonic power of 200 W.

### 3.5. Calculation of the Bagasse CNC Yield

The yield of bagasse CNC was determined by a weighing method using the following equation:

(1) $$y(\%)=\frac{m_{1}}{m_{2}}\times 100\%,$$

where y is the CNC yield of the bagasse (%), *m*_1_ is the CNC mass after freeze-drying (g), and *m*_2_ is the added bagasse crude cellulose mass (g).

### 3.6. Optimization of the Preparation Process Conditions by Orthogonal Test

Based on the single-factor test, orthogonal tests were conducted on four factors, namely acid-digestion temperature, acid-digestion time, sulfuric acid volume fraction, and ultrasonic power, at three levels each, and the bagasse CNC yield was used as the index for investigation. The results of the orthogonal tests were reasonably analyzed and certified to determine the optimal process conditions for the preparation of the bagasse CNCs. Without considering the interactions between the factors, an L9(3^4^) orthogonal table was selected for the test, and the test was repeated twice at each test site. The coding table of the orthogonal factor levels is shown in Table 4.

### 3.7. Surface Functionalization Modification

#### 3.7.1. CNC Bagasse Modified with L-Malic Acid

The L-malic-acid-modified sugarcane bagasse CNCs employed the method of Liang Tao et al., with slight modifications [21]. A certain mass of sugarcane bagasse CNC powder was taken in a three-mouth flask, ultrasonically dispersed with distilled water for 10 min, heated to 110 °C in an oil bath, 10 times the amount of L-malic acid was added, and the heating reaction was continued for 8 h. After 8 h, the unreacted L-malic acid was removed by centrifugal washing with distilled water, and the L-malic-acid-modified nanocellulose (L-CNC) was obtained by vacuum freeze-drying.

#### 3.7.2. Modification of the Sugarcane Bagasse CNCs with the Silane Coupling Agent KH-550

The silane coupling agent KH-550 was used to modify sugarcane bagasse CNCs employing Zhang Xiaoxiao’s method, with slight modifications [22]. We added 0.64 mL of the silane coupling agent KH-550 to 400 mL of distilled water and placed the mixture on a magnetic stirrer for 30 min to fully hydrolyze the KH-550, and then 0.6 g of CNC powder was added. The mixed solution was placed in an ultrasonic cleaner and sonicated for 45 min at 1000 W of power after stirring well, followed by centrifugal washing with distilled water to remove the unreacted silane coupling agent. We then vacuum-freeze-dried the solution to obtain the silane coupling agent KH-550-modified sugarcane bagasse CNCs (K-CNCs).

#### 3.7.3. L-Malic Acid Modified K-CNC

We took a certain mass of K-CNC powder in a three-neck flask and added it to distilled water for 10 min of ultrasonic dispersion, heated it to 110 °C in an oil bath, added 10 times the amount of L-malic acid, and continued the heating reaction. After 8 h, we removed the unreacted L-malic acid by centrifugal washing with distilled water and vacuum-freeze-dried the solution to obtain the L-malic-acid-modified nanocellulose (L-K-CNC).

#### 3.7.4. Modification of the L-CNC with the Silane Coupling Agent KH-550

We took 0.64 mL of the silane coupling agent KH-550 and added it to 400 mL of distilled water, put it on a magnetic stirrer for 30 min to fully hydrolyze the KH-550, added 0.3 g of L-CNC powder, stirred the solution well, and then put the mixed solution in an ultrasonic cleaner and sonicated it for 45 min at 1000 W of power. Then, we washed it with distilled water by centrifugation to remove the unreacted silane. The unreacted silane coupling agent was removed by centrifugation with distilled water, and the bagasse CNC modified with the silane coupling agent KH-550 (K-L-CNC) was obtained by vacuum-freeze-drying.

### 3.8. Analysis and Characterization

#### 3.8.1. Transmission Electron Microscopy Analysis (TEM)

Transmission electron microscopy (JEM-2100 plus-type transmission electron microscope, Nippon Electron Co., Musashino, Japan) was used to observe the surface functions, apparent morphologies, and sizes of the prepared bagasse CNCs compared to the purchased CNCs.

#### 3.8.2. FTIR Analysis

The FTIR (Niolet iN10 FTIR Spectrometer, Thermo Fisher Scientific Co., Waltham, MA, USA) spectra were analyzed by a method that used potassium bromide pressed tablets. We ground a certain amount of sample into powder, and 20 mg of that sample powder was mixed with potassium bromide powder and pressed into tablets. Then, the infrared spectra of the samples were measured in the frequency range of 4000~500 cm^−1^, with a resolution of 4 cm^−1^.

#### 3.8.3. X-ray Diffraction Analysis (XRD)

Using an X-ray diffractometer (Ultima-IV X-ray diffractometer, Rigaku Corporation, Japan), the sample was placed on a sample plate with a Cu Kα copper target (λ = 0.15406 nm) at a power of 1600 W (40 kV × 40 mA), and the scanning range used in this test was 5° to 50°, with a scanning speed of 2° per min.

The degree of crystallinity can be expressed by the crystallinity index (CI), which is given by the following equation:

(2) $$CI(\%)=\frac{I_{200}-I_{am}}{I_{200}}\times 100\%,$$

where *CI* (%) is the crystallinity index; *I*_200_ is the diffraction peak intensity in the crystalline region, 2θ = 22.5°, and *I*_am_ is the diffraction peak intensity in the amorphous region, which is 2θ = 18°.

#### 3.8.4. Thermogravimetric Analysis (TGA)

At room temperature, 5–10 mg of the dried sample was weighed and tested under nitrogen (N_2_) conditions with a nitrogen flow rate of 50 mL/min and a heating rate of 10 °C/min in the temperature range of 30–600 °C (TGA/DSC-1 Synchronous Thermal Analyzer, METTLER TOLEDO International Co., Zurich, Switzerland).

### 3.9. Data Processing

We repeated each experiment 3 times and took the average value. The data processing and analyses were conducted using Excel 365, SPSS 25.0, and Origin 2019b.

## 4. Conclusions

On the basis of the single-factor test, orthogonal tests were conducted on four factors, namely acid-digestion temperature, acid-digestion time, sulfuric acid volume fraction, and ultrasonic power, at three levels each, and the results of the orthogonal tests were reasonably analyzed and certified. Finally, it was concluded that an acid-digestion temperature of 50 °C, an acid-digestion time of 70 min, a sulfuric acid volume fraction of 55%, and an ultrasonic power of 250 W were the optimal process conditions. The modified CNCs were analyzed using infrared spectral, X-ray diffraction, and thermogravimetric techniques, and the modified K-L-CNC showed the best structure and performance. The results of this experiment can provide a reference for the high-value utilization of bagasse, a byproduct of sugar cane production, and the broadening of the comprehensive utilization of bagasse.

## Figures and Tables

**Table 1:** Single-factor orthogonal test results and analysis of the extreme differences.

Test Serial No.	Factors				Repeat Test Indexes		Indicator Addition
	A	B	C	D	1	2	
1	1	1	1	1	23.125	22.220	45.345
2	1	2	2	2	21.455	20.105	41.560
3	1	3	3	3	19.485	17.230	36.715
4	2	1	2	3	27.365	26.440	53.805
5	2	2	3	1	22.420	21.495	43.915
6	2	3	1	2	27.550	26.460	54.010
7	3	1	3	2	19.535	19.990	39.525
8	3	2	1	3	23.615	23.180	46.795
9	3	3	2	1	24.450	22.645	47.095
K1	123.620	138.675	146.150	136.355			
K2	151.730	132.270	142.460	135.095			
K3	133.415	137.820	120.155	137.315			
k1	20.603	23.113	24.358	22.726			
k2	25.288	22.045	23.743	22.516			
k3	22.236	22.970	20.026	22.886			
Polar difference R	4.685	1.068	4.333	0.370			
Superior level	A_2_	B_1_	C_1_	D_3_			

**Table 2:** Analysis of variance for the results of the one-way orthogonal test.

Variance Source	SS	f	MS	F	Level of Significance
Factor A	67.864	2	33.932	42.771	α = 0.01
Factor B	4.031	2	2.015	2.540	Not significant
Factor C	65.937	2	32.969	41.557	α = 0.01
Factor D	0.413	2	0.207	0.260	Not significant
Errors E	7.140	9	0.793		
Sum T	145.386	17			

Notes: F_0.01_ (2, 9) = 8.02; F_01_ (2, 9) = 3.01.

**Table 3:** Analysis of variance after combining errors for the results of one-way orthogonal tests.

Variance Source	SS	f	MS	F	Level of Significance
Factor A	67.864	2	33.932	49.392	α = 0.01
Factor B	4.031	2	2.015	2.933	α = 0.1
Factor C	65.937	2	32.969	47.990	α = 0.01
Errors E	7.553	11	0.687		
Sum T	145.386	17			

Notes: F_0.01_ (2, 11) = 7.20; F_0.1_ (2, 11) = 2.86.

**Table 4:** Table of orthogonal factor levels.

Level	Factors			
	A (Acidification Temperature) (℃)	B (Acidification Time) (min)	C (Sulfuric Acid Volume Fraction) (%)	D (Ultrasound Power) (W)
1	45	70	55	200
2	50	90	60	250
3	55	110	65	300

## Data Availability

The data included in the article are referenced in the article.

## Additional Information

No additional information is available for this paper.

## Sample Availability

Samples of the compounds are available upon request.
